# The CO_2_ Absorption in Flue Gas Using Mixed Ionic Liquids

**DOI:** 10.3390/molecules25051034

**Published:** 2020-02-25

**Authors:** Guoqing Wu, Ying Liu, Guangliang Liu, Xiaoying Pang

**Affiliations:** College of Chemistry and Chemical Engineering, Inner Mongolia University, Hohhot 010021, China; 15771380268@163.com (G.W.); 111975317@imu.edu.cn (G.L.); Pmmwhydedabai@163.com (X.P.)

**Keywords:** flue gas, carbon dioxide, absorption, ionic liquids

## Abstract

Because of the appealing properties, ionic liquids (ILs) are believed to be promising alternatives for the CO_2_ absorption in the flue gas. Several ILs, such as [NH_2_emim][BF_4_], [C_4_mim][OAc], and [NH_2_emim[OAc], have been used to capture CO_2_ of the simulated flue gas in this work. The structural changes of the ILs before and after absorption were also investigated by quantum chemical methods, FTIR, and NMR technologies. However, the experimental results and theoretical calculation showed that the flue gas component SO_2_ would significantly weaken the CO_2_ absorption performance of the ILs. SO_2_ was more likely to react with the active sites of the ILs than CO_2_. To improve the absorption capacity, the ionic liquid (IL) mixture [C_4_mim][OAc]/ [NH_2_emim][BF_4_] were employed for the CO_2_ absorption of the flue gas. It is found that the CO_2_ absorption capacity would be increased by about 25%, even in the presence of SO_2_. The calculation results suggested that CO_2_ could not compete with SO_2_ for reacting with the IL during the absorption process. Nevertheless, SO_2_ might be first captured by the [NH_2_emim][BF_4_] of the IL mixture, and then the [C_4_mim][OAc] ionic liquid could absorb more CO_2_ without the interference of SO_2_.

## 1. Introduction

The CO_2_ absorption of flue gas is an important process for reducing greenhouse gas [1]. To date, most flue absorptions are performed by using amine solvents. However, conventional absorption methods usually have some disadvantages: High equipment corrosion rate, high absorbent make-up rate due to the amine degradation by SO_2_, NO_2_, and O_2_ in the flue gas, and high energy consumption during the regeneration process [2]. In the last decades, ionic liquids (ILs) have been used in many fields. Multifunctional ionic liquids are easily prepared, and the vapor pressure of ionic liquids can be neglected [3]. The other attractive properties of ILs include: High thermal stability, large electrochemical window, and high dissolve ability of compounds [4]. Blanchard et al. [5] have reported that certain ILs can considerably dissolve CO_2_ gas. Since then, ILs for CO_2_ capture have attracted much attention. For example, multi-*N*-containing ionic liquids can absorb much more SO_2_/CO_2_ in the flue gas than that of the limestone solvent [6]. Shiflett et al. [7,8] have found that the imidazolium-based ionic liquid [C_4_mim][OAc] can markedly reduce the energy losses of CO_2_ absorption comparing with those of the commercial monoethanolamine solvent. Guanidinium salt ILs (e.g., [TMG][L]) and functional ILs (e.g., [NH_2_p-bmim][BF_4_] and 2-(2-hydroxyethoxy) ammonium acetate) all show high efficiency for CO_2_ and SO_2_ capture [9,10].

The typical flue gas from coal-burning usually contains about 15 vol% CO_2_, 10 vol% H_2_O, and more than 2 vol% SO_2_ [11]. Apart from CO_2_, the effects of SO_2_ on the flue gas absorption should be taken into account [12]. Most researchers consider that water has a little influence on the CO_2_ capture, but the effects of SO_2_ would not be neglected. For the impurities of the flue gas, it is found that the ILs are more likely to absorb SO_2_ than CO_2_. Specifically, the N element of the ionic liquid (IL) prefers to capture the SO_2_ molecules, and then CO_2_ molecules are repelled by the captured SO_2_ [13]. Thus, the CO_2_ absorption capacity of ionic liquids would be rapidly decreased over several cycles. Moreover, almost all of the ILs would exhibit much higher SO_2_ absorption capacity than CO_2_ due to the higher solubility of SO_2_ in ILs. For instance, pure CO_2_ solubility in the guanidinium-based ILs was only 0.4 mol/mol, while the SO_2_ solubility in these ILs was as high as 2.5 mol/mol under the same conditions [10]. For an extreme case, the absorption capacities of pure SO_2_ and CO_2_ in the azole-based ILs (e.g., [P_66614_][Im]) were 3.5 mol/mol and 0.1 mol/mol, respectively [14]. Although SO_2_ has stronger interactions with ILs than CO_2_ does, the actual partial pressure of SO_2_ in the flue gas is very low [15]. It is well accepted that the partial pressure of SO_2_ is about two orders of magnitude lower than that of CO_2_, and low SO_2_ partial pressure usually leads to low absorption capacity for SO_2_. That is, a lot of energy will be consumed to remove SO_2_ and CO_2_ from the flue gas, if we used a two-step absorption process (absorbing SO_2_ at first, and then CO_2_).

The effects of single IL on the CO_2_ capture have been widely discussed in recent years [2,7,16,17,18,19]. However, there are few reports on the IL mixtures for CO_2_ absorption, especially on the CO_2_ capture from the flue gas. Therefore, if the IL mixture was employed, the CO_2_ absorption capacity of the mixed ILs might be greater than that of the single ionic liquid. Specifically, if the SO_2_ of flue gas was absorbed by one IL of the mixture, the negative influence of SO_2_ on the whole IL mixture might be significantly decreased, and then more CO_2_ could be captured.

In this work, we want to use IL mixtures to remove CO_2_ and SO_2_ from the flue gas. The amine-functional ionic liquid [NH_2_emim][BF_4_] and the imidazolium-based ionic liquid [C_4_mim][OAc] were synthesized at first. Subsequently, the two ILs were mixed to investigate the CO_2_ and SO_2_ absorption. Here, [NH_2_emim][BF_4_] was mainly used to absorb SO_2_ in the flue gas, while the [C_4_mim][OAc] ionic liquid was employed to capture CO_2_. In order to more clearly study the actual flue gas, the CO_2_ absorption performance in the IL mixture was measured at the simulated flue gas with 15 vol% CO_2_ and 2 vol% SO_2_. The effect of SO_2_ on the IL mixture and the interaction between CO_2_ and SO_2_ in the IL absorption were also studied. Furthermore, the absorption mechanism at the molecule level was investigated by the quantum chemical calculation and the instrumental analysis.

## 2. Results and Discussion

### 2.1. CO_2_ and SO_2_ Absorption Performance of the Single ILs

When the simulated flue gas (15% CO_2_/85% N_2_) only contains the CO_2_ impurity, one mole [C_4_mim][OAc] could absorb 0.298 mol CO_2_ (Table 1). However, when SO_2_ was mixed in the simulated flue gas (15% CO_2_/2% SO_2_/N_2_), the CO_2_ absorption capacity of [C_4_mim][OAc] was reduced to 0.204 CO_2_/mol IL. The result shows that SO_2_ has a negative effect on the IL absorbing CO_2_. The single IL [NH_2_emim][OAc] and [NH_2_emim][BF_4_] were also used to capture the CO_2_ of flue gas. Without the interference of SO_2_, the CO_2_ absorption capacities of [NH_2_emim][OAc] and [NH_2_emim][BF_4_] were 0.291 mol CO_2_/mol IL and 0.290 mol CO_2_/mol IL, respectively. However, like the [C_4_mim][OAc] ionic liquid, the CO_2_ absorption capacities under exposure to 2% SO_2_ would be markedly decreased to 0.171 mol CO_2_/mol [NH_2_emim][OAc] and 0.180 mol CO_2_/mol [NH_2_emim][BF_4_], respectively. In short, the single ionic liquids all exhibit the CO_2_ absorption capacities, but this capacity would be greatly weakened by the interference of SO_2_.

**Figure 1:**
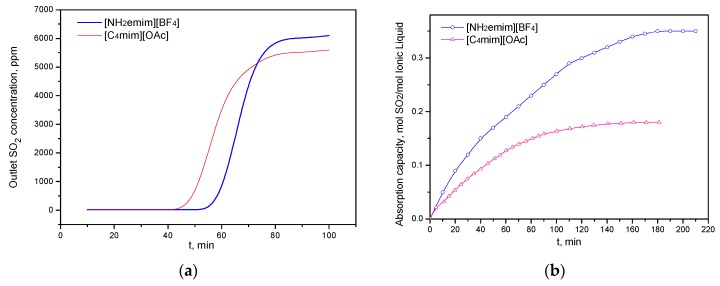
SO_2_ absorption performance of [C_4_mim][OAc] and [NH_2_emim][BF_4_]: (**a**) Outlet SO_2_ concentration vs. time at the atmosphere of 15% CO_2_, 2% SO_2_, and 83% N_2_; (**b**) SO_2_ absorption capacity at the atmosphere of 2% SO_2_/98% N_2_.

The researchers believed that CO_2_ and SO_2_ of the flue gas would be absorbed simultaneously [13,20]. The [C_4_mim][OAc] and [NH_2_emim][BF_4_] absorption results supported this conclusion. For example, an experiment was carried out of outlet SO_2_ concentration vs. time to investigate the SO_2_ absorption performance. In this study (Figure 1a), the simulated flue gas contained 15% CO_2_, 2% SO_2_, and 83% N_2_. The concentrations of CO_2_ and SO_2_ of the outlet stream were simultaneously detected with time. It was found that [C_4_mim][OAc] and [NH_2_emim][BF_4_] all can capture CO_2_, but the outlet SO_2_ concentration was hardly detected before 40 min (Figure 1a). It indicates that SO_2_ could be completely absorbed by [C_4_mim][OAc] and [NH_2_emim][BF_4_] during the absorption process. Additionally, an extreme case was investigated in which the simulated flue gas contained 80% CO_2_, 2% SO_2_, and 18% N_2_. However, SO_2_ was also not found at the gas stream of the outlet before 15 min. Compared with CO_2_, SO_2_ has higher dipole moments and molecular polarity, which often results in the strong affinity of SO_2_ with ionic liquids [21,22].

**Figure 2:**
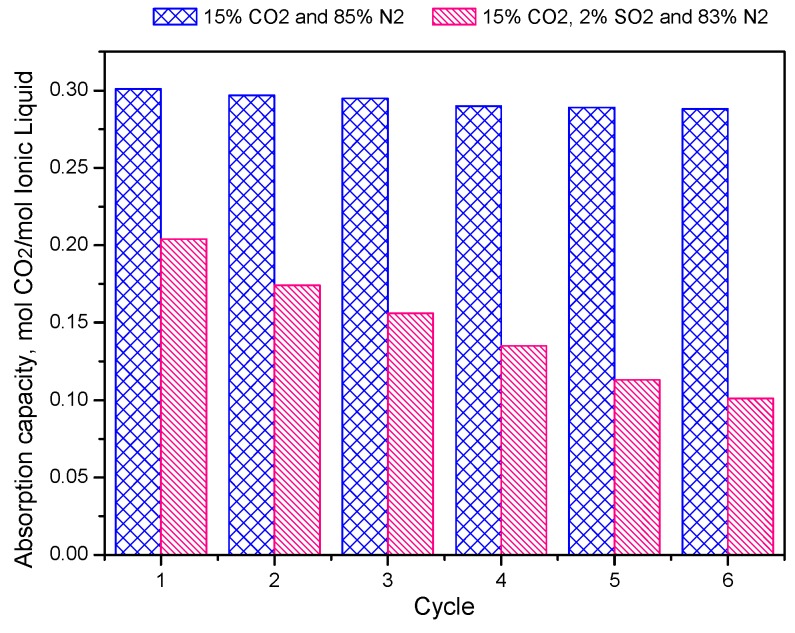
CO_2_ absorption capacity of [C_4_mim][OAc] during 6 regeneration cycles at the atmosphere of 15% CO_2_/85% N_2_ and 15% CO_2_/2% SO_2_/83% N_2_.

The presence of SO_2_ in flue gas usually leads to a competitive and negative influence on the separation of CO_2_. Figure 2 shows the CO_2_ absorption performance of [C_4_mim][OAc] at the atmosphere of 15% CO_2_/85% N_2_ and 15% CO_2_/2% SO_2_/83% N_2_, respectively. After 6 regeneration cycles, 1 mol [C_4_mim][OAc] could absorb 0.255 mol CO_2,_ although the IL absorption capacity slightly decreased. In contrast, the absorption capacity of 1 mol [C_4_mim][OAc] was only 0.10 mol CO_2_ after the same number of cycles. In addition, the net SO_2_ absorption experiment (2% SO_2_/98% N_2_) showed that [NH_2_emim][BF_4_] has high SO_2_ absorption capacity (0.35 mol SO_2_/mol IL), while 1 mol [C_4_mim][OAc] only absorbs 0.18 mol SO_2_ (Figure 1b). These results agreed well with previous studies [23,24,25].

**Figure 3:**
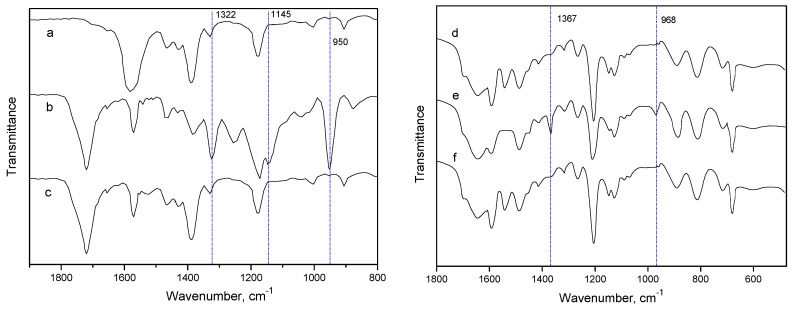
FT-IR spectra of ionic liquids (ILs): (**a**) Fresh [C_4_mim][OAc]; (**b**) [C_4_mim][OAc] after SO_2_ absorption (2% SO_2_/98% N_2_); (**c**) [C_4_mim][OAc] after CO_2_ absorption (15% CO_2_/85% N_2_); (**d**) Fresh [NH_2_emim][BF_4_]; (**e**) [NH_2_emim][BF_4_] after SO_2_ absorption (2% SO_2_/98% N_2_); (f) [NH_2_emim][BF_4_] after CO_2_ absorption (15% CO_2_/85% N_2_).

FT-IR can investigate the interaction between IL and CO_2_/SO_2_ [26]. The spectra of [C_4_mim][OAc] showed the changes after 15% CO_2_ and 2% SO_2_ absorption, respectively (Figure 3a−c). However, the [C_4_mim][OAc] spectrum had minimal changes when it was used to remove pure CO_2_. Although the appearance of the carbonyl band at 1720 cm^−1^ shows that the acetate anion might be partly converted into the acetate acid [27], Shiflett considered that the amount of such a chemical reaction was minor and reversible [24]. Thus, the other reactions between CO_2_ and the cation species in this work might not be detected within the wavenumber of 800−1600 cm^−1^, as Figure 3a,c shown. Similarly, the FT-IR spectra of [NH_2_emim][BF_4_] almost have no change before and after CO_2_ absorption (Figure 3d,f), indicating that the chemical reaction between the [NH_2_emim] cation and CO_2_ was not enough to be detected by FT-IR.

In contrast, the chemical reaction between SO_2_ and [C_4_mim][OAc] was much stronger than that of [C_4_mim][OAc]−CO_2_. Even if there was only a small amount of SO_2_ (2%) in the flue gas, the FT-IR spectrum still shows marked changes (Figure 3b). Compared with the spectrum of fresh [C_4_mim][OAc], the peak intensity at 1580 and 1371 cm^−1^ decreased significantly as the [C_4_mim][OAc] absorbing SO_2_. Meanwhile, the peaks at 1720, 1321, 1254, 1144, and 950 cm^−1^ newly appeared in the spectrum. The bands at 1321 and 1144 cm^−1^ can be attributed to the stretching of SO_2_ absorbed by the ionic liquid [26]. After SO_2_ absorption, the new peak at 1720 cm^−1^ shows the formation of a carbonyl group, which also indicates that most of the acetate ions were no longer associated with [C_4_mim] cations. The intense band at 950 cm^−1^ should be assigned to the vibrational mode of SO_3_^2−^ or S_2_O_5_^2−^. It once again suggests that the interaction between the SO_2_ and [C_4_mim][OAc] ionic liquid was strong. Similarly, the peak intensity at 885 and 1543 cm^−1^ changed markedly when the [NH_2_emim][BF_4_] absorbed SO_2_. Particularly, two new peaks appeared at 968 and1367 cm^−1^, which can be attributed to the interaction between the N elements of the IL and SO_2_ [6].

### 2.2. CO_2_/SO_2_ Absorption Properties in IL Mixtures

**Figure 4:**
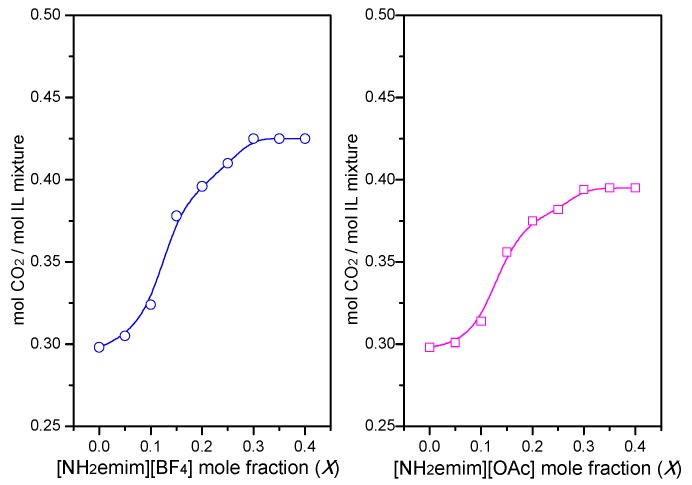
CO_2_ absorption capacity of ionic liquid (IL) mixtures at different *X*: Flue atmosphere 15% CO_2_/2% SO_2_/83% N_2_; absorption temperature, 293 K.

Due to the influence of SO_2_, the CO_2_ absorption capacity of single ionic liquids was greatly decreased. If [NH_2_emim][BF_4_] and [C_4_mim][OAc] were simultaneously utilized, more CO_2_ (with SO_2_) in the flue gas might be captured. The CO_2_ absorption capacities of IL mixtures at different mole fractions of [C_4_mim][OAc] or [NH_2_emim][BF_4_] were displayed in Figure 4. Here, *X* is defined as the molar ratio of [NH_2_emim][BF_4_] to the IL mixture ([C_4_mim][OAc]/[NH_2_emim][BF_4_]). Compared with the CO_2_ absorption capacity of the single IL, the IL mixture could remove more CO_2_. This result may be due to the presence of [NH_2_emim][BF_4_]. The small amount of SO_2_ might be absorbed by [NH_2_emim][BF_4_] at first. Without the interference of SO_2_, the CO_2_ absorption capacity of the IL mixture was significantly enhanced. It was also found that the absorption of CO_2_ did not increase significantly with the increase of the *X* value. When *X* was 0.3, the CO_2_ absorption capacity of the [C_4_mim][OAc]/[NH_2_emim][BF_4_] mixture reached up to the maximum. Similarly, when the IL mixture [C_4_mim][OAc]/[NH_2_emim][OAc] was used to remove CO_2_/SO_2_ of the flue gas, the poison effect of SO_2_ on [C_4_mim][OAc] was also greatly reduced. As Figure 4 shows, the CO_2_ absorption of [C_4_mim][OAc]/[NH_2_emim][OAc] was about 0.4 mol CO_2_/mol IL. Compared to the single [C_4_mim][OAc], the absorption capacity of the IL mixture was improved.

**Figure 5:**
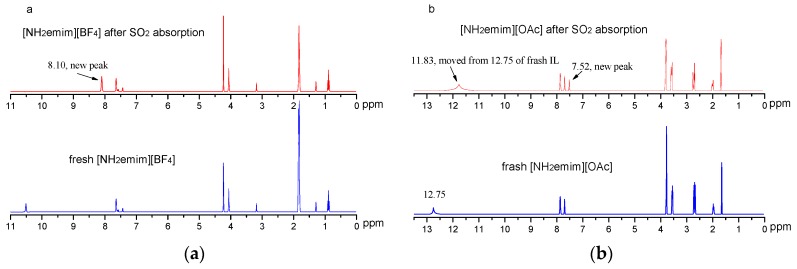
^1^H NMR spectra of ILs: (**a**) [NH_2_emim][BF_4_]; (**b**) [NH_2_emim][OAc].

The ^1^H NMR spectrum (Figure 5) shows that SO_2_ would interact with [NH_2_emim][BF_4_] and [NH_2_emim][OAc]. The NMR data of fresh [NH_2_emim][BF_4_] and fresh [NH_2_emim][OAc] are listed as follows:

Fresh [NH_2_emim][BF_4_], *δ* = 7.721 (s, 1H, unsaturated C−H in the imidazole ring, with N connected to the left and right), 7.641 (d, 1H, unsaturated C−H in the imidazole ring), 7.636 (d, 1H, unsaturated C−H in the imidazole ring), 4.295 (s, 3H, H_3_C−N ring), 4.039 (t, 2H, H_2_C−N ring), 3.112 (m, 2H, N−CH_2_−C−N ring), and 1.878 (t, 2H, NH_2_).

Fresh [NH_2_emim][OAc], *δ* = 12.751 (s, 1H, unsaturated C−H in the imidazole ring, with N connected to the left and right), 7.865 (d, 1H, unsaturated C−H in the imidazole ring), 7.703 (d, 1H, unsaturated C−H in the imidazole ring), 3.577 (s, 3H, H_3_C−N ring), 3.749 (t, 2H, H_2_C−N ring), 2.693 (m, 2H, N−CH_2_−C−N ring), 1.973 (t, 2H, NH_2_) and 1.651 (s, 3H, CH_3_ in OAc-).

In comparison to the ^1^H NMR spectrum of the fresh [NH_2_emim][BF_4_] (Figure 5a), new resonance peaks at 8.10 ppm were found after SO_2_ absorption, which indicates the formation of S^…^N [28]. According to this result, it was considered that the interaction between SO_2_ and [NH_2_emim][BF_4_] should mainly occur at the N element of the [NH_2_emim] cation. For the case of the [NH_2_emim][OAc] (Figure 5b), a typical peak of −COOH in the ^1^H NMR spectrum moved from 12.75 to 11.83 ppm, and a new resonance peak was observed at 7.52 ppm after SO_2_ absorption. These results suggest that the interaction between [NH_2_emim][OAc] and SO_2_ had occurred [25]. That is, the interaction between [OAc] and SO_2_ leads to the moving from 12.75 ppm to 11.83, while the reaction of [NH_2_emim] and SO_2_ makes the new peak 7.52 ppm appearance.

**Figure 6:**
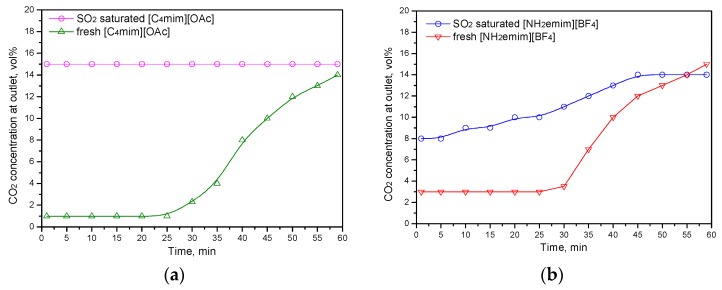
CO_2_ absorption performance of fresh IL and after SO_2_ saturated IL: (**a**) [C_4_mim][OAc]; (**b**) [NH_2_emim][BF_4_].

In order to further investigate the effects of SO_2_ on the CO_2_ absorption capacity of ionic liquids, the CO_2_ absorption performance of fresh IL and after SO_2_-saturated IL are illustrated in Figure 6. Specifically, fresh [C_4_mim][OAc] and fresh [NH_2_emim][BF_4_] ionic liquids were used to absorb SO_2_ at first. When the IL was saturated by SO_2_, the CO_2_ absorption performance of the IL was investigated. It was found that the SO_2_-saturated [C_4_mim][OAc] did not have the ability to absorb CO_2_. The concentration of CO_2_ at the outlet was almost equal to that at the inlet. While fresh [C_4_mim][OAc] can absorb CO_2_ even after 60 min. The similar results were also observed using fresh [C_2_mim][OAc] and SO_2_ saturated [C_2_mim][OAc] to absorb CO_2_ [24]. Shiflet et al. considered that the interaction between the [OAc] anion and CO_2_ plays an important role in the CO_2_ removal of [OAc]−based ionic liquids [8]. However, the presence of SO_2_ makes a great impact on the CO_2_ absorption of [C_4_mim][OAc]. In contrast, when [NH_2_emim][BF_4_] was saturated by SO_2_, the [NH_2_emim][BF_4_] still had the CO_2_ absorption capacity. As Figure 6b shows, SO_2_-saturated [NH_2_emim][BF_4_] could capture about 2−7% CO_2_ during the absorption process.

### 2.3. Quantum Chemical Calculation on the Interaction of IL Mixture with CO_2_/SO_2_

**Figure 7:**
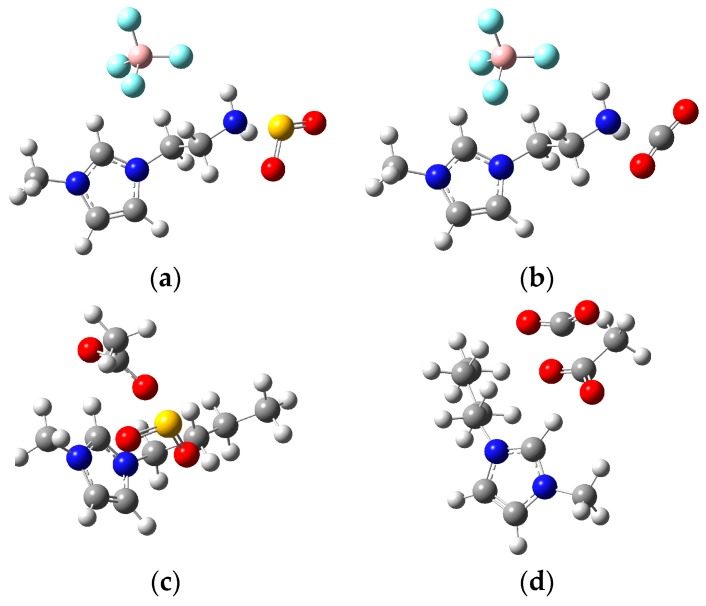
Optimized structure of the complexes of IL with CO_2_/SO_2_: (**a**) [NH_2_emim][BF_4_]−SO_2_; (**b**) [NH_2_emim][BF_4_]−CO_2_; (**c**) [C_4_mim][OAc]−SO_2_; (**d**) [C_4_mim][OAc]−CO_2_.

The absorption capacity of CO_2_ in the IL mixtures was higher than that of the single ionic liquid. This may be related to the interactions between ILs and CO_2_/SO_2_ molecules. Thus, the interaction of the [C_4_mim][OAc] anion and [NH_2_emim][BF_4_] with CO_2_/SO_2_ was deeply investigated through quantum chemical calculation, which might be helpful to understand well the roles of CO_2_ and SO_2_ in the IL absorption. In this work, the structure of [NH_2_emim][BF_4_] and [C_4_mim][OAc] was optimized on the basis of DFT-D3 calculation at first. The configuration of the IL with the lowest energy was considered as the optimized structure. Additionally, the structures of [C_4_mim][OAc] and [NH_2_emim][BF_4_] with CO_2_ and SO_2_ were also investigated (Figure 7). The structural parameters for the IL−CO_2_/SO_2_ complexes are listed in Table 2.

In general, the CO_2_/SO_2_ gas molecules around the anions and cations were related to the absorption reaction. As Figure 7a shows, there was a strong interaction between the N atom and the S atom of SO_2_. Due to the complexation of SO_2_^…^N, the average angle of SO_2_ was 116.0°. Compared to 119.5° of the pure SO_2_ molecule, the bending degree of O=S=O was increased. Similarly, [NH_2_emim][BF_4_] also leads to an impact on the CO_2_ structure. The angle of CO_2_ was bent from 180° to 166°, and the bond length of C−O was extended from 1.16 Å to 1.19 Å. For the cases of [C_4_mim][OAc], the interaction between the O and the C atom of carbon dioxide was also strong due to the negatively charged oxygen (O atom) in the [OAc] anion. The curvature of CO_2_ could be increased by the interaction of the [OAc] anion. The average angle of CO_2_ was bent to 146°, and the bond length of C−O was elongated to 1.21 Å. The configurations in Figure 7 also suggest that [NH_2_emim] cation and [OAc] anion are the active sites for CO_2_/SO_2_ absorption.

To some extent, the interaction energy and absorption enthalpy might reflect the absorption capacity of the ILs. It was found that the NH_2_emim] cation and [OAc] anion were the main active sites for the absorption of CO_2_ and SO_2_. In order to save the calculation cost and reduce the interference of other ions, herein, only the thermodynamic data of [OAc]−CO_2_, [OAc]−SO_2_, CO_2_−[NH_2_emim], and SO_2_−[NH_2_emim] complexes were compared (Table 3). It was found that the interaction energy and absorption enthalpy of [OAc]−CO_2_−SO_2_ complex were less than the sum of the energy and the enthalpy for [OAc]−CO_2_ and [OAc]−SO_2_, suggesting that CO_2_ and SO_2_ would competitively react with [OAc] anion.

In contrast, the interaction energy and absorption enthalpy of [NH_2_emim]−CO_2_−SO_2_ complex were approximately equal to the sum of those for the [NH_2_emim]−CO_2_ and [NH_2_emim]−SO_2_ complexes, indicating that the competitive reaction between [NH_2_emim]−CO_2_ and [NH_2_emim]−SO_2_ was not obvious. The absorption reaction might also lead to a change in charge distribution. It was found that the amount of net charge transfer from CO_2_ to [NH_2_emim] in [NH_2_emim]−CO_2_−SO_2_ complex was almost equal to that of [NH_2_emim]−CO_2_, suggesting that [NH_2_emim] had strong interactions with either SO_2_ or CO_2_. However, due to the impact of SO_2_, the net charge transfer from [OAc] to CO_2_ was significantly reduced from −0.510 in the [OAc]−CO_2_ complex to −0.035 in [OAc]−CO_2_−SO_2_ complex, respectively, which might account for the decrease of CO_2_ absorption capacity for [C_4_mim][OAc] in Table 1.

Table 4 collects the thermochemical data of [NH_2_emim][BF_4_]−CO_2_ and [NH_2_emim][BF_4_]−SO_2_ complexes. In order to consider the solvent effect of ionic liquids, the continuum universal solvation model (SMD) was used in the calculation. Based on the SMD model, the interaction energies of [NH_2_emim][BF_4_]−CO_2_ and [NH_2_emim][BF_4_]−SO_2_ system were −16.8 and −76.3 kJ/mol, respectively. They were lower than those in the gas phase (−19.2 and −85.4 kJ/mol). Notably, the difference between the interaction energy ([NH_2_emim][BF_4_]−CO_2_ and [NH_2_emim][BF_4_]−SO_2_) in the gas phase (59.5 kJ/mol) was very consistent with the energy difference (66.2 kJ/mol) using the SMD model. In the liquid phase, the interaction energy between [NH_2_emim][BF_4_] and SO_2_ was slightly greater than that of [NH_2_emim][BF_4_]−CO_2_, suggesting that [NH_2_emim][BF_4_] tends to react with SO_2_ rather than with CO_2_ during the absorption process. Similarly, this phenomenon could also be observed in the thermodynamic data of the [C_4_mim][OAc]−CO_2_ and [C_4_mim][OAc]−SO_2_ complexes. In short, SO_2_ was more active than CO_2_ in the reaction with ionic liquids, and the [NH_2_emim][BF_4_] may be more likely to absorb SO_2_.

**Figure 8:**
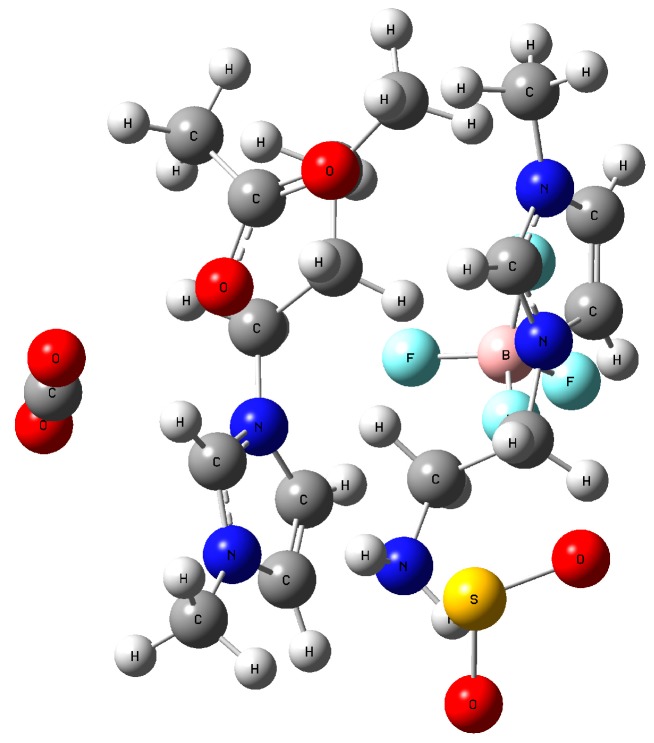
Optimized structure of the IL mixture and CO_2_/SO_2_.

The interaction between IL mixture ([C_4_mim][OAc]/[NH_2_emim][BF_4_]) and CO_2_/SO_2_ has also been investigated by the quantum chemistry calculation. The optimized structure is displayed in Figure 8. In the mixed ionic liquids, it is found that SO_2_ was close to [NH_2_emim][BF_4_], while the CO_2_ molecule was near the [C_4_mim][OAc] ionic liquid. Specifically, SO_2_ would have interacted with the N atom on the [NH_2_emim] cation, and CO_2_ was more likely to react with the [OAc] anion. This result might explain why the IL mixture can more effectively absorb the CO_2_ of flue gas. Because of the existence of [NH_2_emim][BF_4_], SO_2_ may be first captured by [NH_2_emim]. Without the interference of SO_2_, the [C_4_mim][OAc] ionic liquid then could absorb more CO_2_.

## 3. Materials and Methods

### 3.1. Materials

The simulated flue gas was obtained by pure gas CO_2_, SO_2_, and N_2_ (purity of >99.99 wt%). They were all purchased from Beifen (China) Gas Technology Company. 1-butyl-3-methylimidazolium tetrafluoroborate ([C_4_mim][BF_4_], 99 wt%) was obtained from Sigma-Aldrich Chemical Co., but the ionic liquid 1-butyl-3-methylimidazolium acetate ([C_4_mim][OAc], 99 wt%) were purchased from Lanzhou Greenchem ILs, LICP, CAS, China. Additionally, [NH_2_emim][BF_4_] and [NH_2_emim][OAc] have been synthesized by ourselves in this work. The used materials were as follows: 1-methylimidazole (C_4_H_6_N_2_), 2-bromoethylamine hydrobromide (C_2_H_7_Br_2_N), sodium acetate anhydrous (CH_3_COONa), 1-methylimidazole (C_4_H_6_N_2_), acetic acid (CH_3_COOH), and sodium borate (NaBF_4_). They were all provided by Sinopharm Chemical Reagent Co., Ltd., China, with purity over 98 wt%.

### 3.2. Ionic Liquid Preparation

In this work, the ionic liquids [NH_2_emim][BF_4_] and [NH_2_emim][OAc] were prepared by ourselves according to the method used in the literature [17,18,29]. First, the [NH_2_emim] cation was prepared by the reaction of 1-methylimidazole and 2-bromoethylamine hydrobromide under reflux for 12 h. Second, the [NH_2_emim]-based IL was simply synthesized by ion exchange with NaBF_4_ or NaOAc/CH_3_COOH in ethanol, and then the ethanol was removed in vacuum. The structures of the ILs were confirmed by proton nuclear magnetic resonance (^1^H NMR, Bruker WB400 AMX spectrometer, Billerica, MA, USA). Here, deuterated chloroform (CDCl_3_) was used as a solvent, and tetramethylsilane (TMS) was employed as an internal standard for ^1^H NMR measurement.

### 3.3. CO_2_ and SO_2_ Absorption

**Figure 9:**
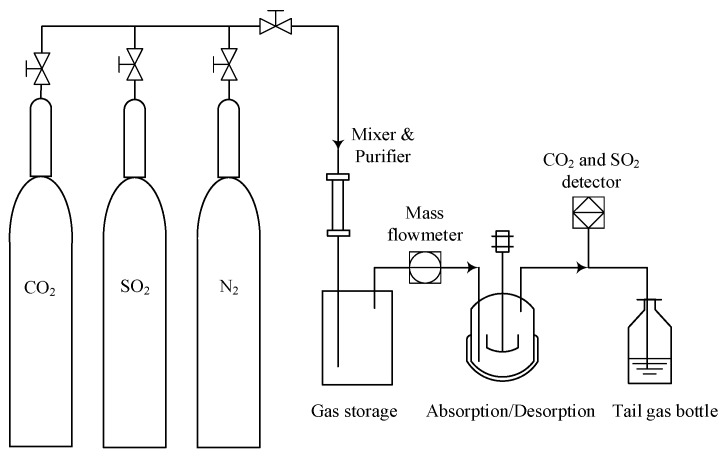
Schematic diagram of the apparatus used for absorption and desorption.

As Figure 9 shows, CO_2_ and SO_2_ absorption experiments were performed in a 30 mL reactor immersed with a water-bath temperature controller. The temperatures were controlled at 293 K for absorption and 353 K for desorption, respectively. The simulated flue gas was a mixture of N_2_, CO_2_, and SO_2_ in accordance with a certain proportion. As a typical absorption process, 10 mL IL or IL mixtures were added to the reactor at first. Subsequently, 15 vol% CO_2_, 2 vol% SO_2_ and 83 vol% N_2_ were mixed in storage. The intake speed of the mixed gas was controlled at 60 mL/min, and the absorption pressure was controlled at 101.3 kPa. The concentrations of CO_2_ and SO_2_ were analyzed by a gas analyzer (MRU NOVA2000) at the outlet. To investigate the IL regeneration, CO_2_ or SO_2_ saturated IL was also loaded in the reactor. Desorption was performed at 353 K under a pure N_2_ gas atmosphere for 30 min.

The amount of absorbed CO_2_ or SO_2_ was calculated by the following equation.

$$A_{\mathrm{gas}}=\frac{M_{\mathrm{IL}}\rho_{\mathrm{gas}}Q\int_{t_{1}}^{t_{2}}\left(C_{0}-C_{\mathrm{gas}}\left(t\right)\right)dt}{m_{\mathrm{IL}}M_{\mathrm{gas}}}$$

where *A*_gas_ is the molar amount of CO_2_ or SO_2_ in the ionic liquid; *Q* is the flow rate of the gas stream; *C*_0_ and *C*_gas_ are the CO_2_ or SO_2_ concentrations at the inlet and the outlet streams, respectively; *t*_1_ refers to the beginning time of the absorption process; when the CO_2_ and SO_2_ concentration at the outlet stream returns to the initial concentration, the time is *t*_2_; *M*_IL_ and *M*_gas_ are the molecular weight of IL and CO_2_ (or SO_2_), respectively; *m*_IL_ is the weight of the ILs, and *ρ*_gas_ is the density of CO_2_ or SO_2_.

After the IL was saturated by CO_2_ and SO_2_, the complex structure was investigated by the FTIR and NMR technologies. The FTIR spectra of the samples were analyzed on an FTIR spectrometer (PerkinElmer, Frontier 2500). In addition, the structure changes of the [NH_2_emim]-based IL after absorption were also detected by the NMR spectrometer (Bruker WB400 AMX, 300 MHz) using chloroform-*d* (CDCl_3_) as a solvent and tetramethylsilane (TMS) as an internal standard.

### 3.4. Theoretical Calculation

A quantum chemical calculation was used in this work to study the interaction between the ionic liquid and CO_2_ with SO_2_. All calculations were carried out by the Gaussian 16 program [30]. For IL calculations, Li et al. [31] suggested that the density function of the Minnesota family [32] (e.g., M06-2X) with a diffusion function basis set (e.g., 6-311++G(d,p)) might give reasonable results. If dispersion-corrected density functionals (e.g., gd3bj, DFT-D3) were used, more reliable results could be obtained [33]. Therefore, the geometry optimization and frequency analysis of all ILs and IL mixtures were performed at the M06-2X/6-311++G(d,p) level and correction with Grimme’s method. In order to calculate the interaction energy of the IL complexes, the basis set superposition error (BSSE) method was employed to correct the energy results [34]. The effect of the solvent should be taken into consideration in the theoretical calculation of the ionic liquids. It was found that the SMD solvation model proposed by Truhlar et al. can be used for the IL calculation very well [35,36]. Thus, the density functional theory (M06-2X and dispersion-corrected method) with the SMD model was also used to calculate the interaction energy of IL−CO_2_/SO_2_.

## 4. Conclusions

The CO_2_ and SO_2_ absorption of the flue gas in ionic liquids were investigated by the experimental method and theoretical calculation. The single ionic liquids, such as [NH_2_emim][BF_4_] and [C_4_mim][OAc], all showed good CO_2_ absorption performance for the simulated flue gas without SO_2_ interference. However, SO_2_ was more likely to react with the active sites of the ILs. When SO_2_ was in the flue gas, the CO_2_ absorption capacity of the single ionic liquid would be significantly inhibited. It was found that the interference of SO_2_ on the CO_2_ absorption performance might be markedly reduced by using the ionic liquid mixtures. The CO_2_ absorption capacity of the IL mixture [C_4_mim][OAc]/[NH_2_emim][BF_4_] was about 0.4 mol CO_2_/mol IL even at an atmosphere of 15% CO_2_/2%SO_2_/83% N_2_, which was greater than that of single [C_4_mim][OAc] (0.204 mol CO_2_/mol IL). There was a competitive relationship between CO_2_ and SO_2_ during the absorption process. The single ILs prefer to capture SO_2_ rather than remove CO_2_, due to the stronger interaction energy of SO_2_ and the ILs. The experimental and calculated results suggested that the [OAc] anion and [NH_2_emim] cation are the main active sites for CO_2_ and SO_2_ absorption. A lower absorption enthalpy of the IL−SO_2_ or IL−CO_2_ system usually means low absorption capacity. Thus, for the IL mixture [C_4_mim][OAc]/[NH_2_emim][BF_4_], the quantum calculation results indicated that [NH_2_emim][BF_4_] might be more likely to absorb SO_2_ of the flue gas and CO_2_ was easily removed by the [C_4_mim][OAc].

## Figures and Tables

**Table 1:** Summary of CO_2_ absorption capacity by single ionic liquids.

Ionic Liquids	T, *K*	Gas	Absorption Capacity, mol CO_2_/mol IL
[C_4_mim][OAc]	293	15% CO_2_/85% N_2_	0.298
[C_4_mim][OAc]	293	15% CO_2_/2% SO_2_/83% N_2_	0.204
[NH_2_emim][BF_4_]	293	15% CO_2_/85% N_2_	0.290
[NH_2_emim][BF_4_]	293	15% CO_2_/2% SO_2_/83% N_2_	0.180
[NH_2_emim][OAc]	293	15% CO_2_/85% N_2_	0.291
[NH_2_emim][OAc]	293	15% CO_2_/2% SO_2_/83% N_2_	0.171

**Table 2:** Structural parameters for the complexes.

Structural Parameters	CO_2_−[NH_2_emim][BF_4_]	SO_2_−[NH_2_emim][BF_4_]	[C_4_mim][OAc]−CO_2_	[C_4_mim][OAc]−SO_2_
C−O, Å	1.19		1.21	
∠O−C−O, °	166		146	
S−O, Å		1.46		1.49
∠O−S−O, °		113.5		112.6

**Table 3:** Thermochemical parameters and charge transfer of the ion−CO_2_/SO_2_ complexes.

	**OAc−CO_2_**	**OAc−SO_2_**	**OAc−CO_2_−SO_2_**
*Δ*E, kJ/mol	−40.7	−113.4	−140.0
*Δ*H, kJ/mol	−46.5	−125.1	−151.4
*Δ*G, kJ/mol	−1.6	−70.8	−72.1
net charge transfer, e	−0.510	−0.382	−0.035(CO_2_)/−0.316(SO_2_)
	**CO_2_−[NH_2_emim]**	**SO_2_−[NH_2_emim]**	**SO_2_−CO_2_−[NH_2_emim]**
*Δ*E, kJ/mol	−33.8	−123.9	−156.8
*Δ*H, kJ/mol	−36.3	−126.7	−160.1
*Δ*G, kJ/mol	−7.2	−55.1	−61.9
net charge transfer, e	−0.312	−0.399	−0.308(CO_2_)/−0.334(SO_2_)

**Table 4:** Thermochemical parameters for the IL−CO_2_/SO_2_ complexes ^a^.

	[C_4_mim][OAc]−CO_2_	[C_4_mim][OAc]−SO_2_	CO_2_−[NH_2_emim][BF_4_]	SO_2_−[NH_2_emim][BF_4_]
*Δ*E, kJ/mol	−26.4 (−21.9)	−80.1 (−70.5)	−19.2 (−16.8)	−85.4 (−76.3)
*Δ*H, kJ/mol	−30.5	−93.2	−29.1	−91.5
*Δ*G, kJ/mol	−2.7	−40.6	8.1	−37.2

^a^ Values of brackets were calculated by the continuum universal solvation model (SMD).
